# 81 Correction of bias in self-reported 24-hour movement behaviours: How well can the bias be removed?

**DOI:** 10.1093/eurpub/ckae114.051

**Published:** 2024-09-26

**Authors:** Kaja Kastelic, Marija Rakić, Anja Šuc, Nejc Šarabon

**Affiliations:** University of Primorska, Andrej Marušič Institute, Koper, Slovenia; InnoRenew CoE, Izola, Slovenia; University of Primorska, Faculty of Mathematics, Natural Sciences and Information Technologies, Koper, Slovenia; University of Primorska, Faculty of Health Sciences, Izola, Slovenia; InnoRenew CoE, Izola, Slovenia; University of Primorska, Faculty of Health Sciences, Izola, Slovenia

## Abstract

**Background:**

Measurement of self-reported 24-hour movement behaviours (i.e. physical activity, sedentary behaviour, and sleep) is important for research, policy, and practice. However, studies showed that self-reported estimates are substantially biased, and that bias usually differs by individual’s characteristics (e.g. age, sex, body mass index). This study aimed to calibrate a recently developed questionnaire for the assessment of moderate-to-vigorous physical activity (MVPA), light physical activity (LPA), sedentary behaviour (SB), and sleep; and to explore how well can the bias be removed.

**Methods:**

We pooled micro-level data from previous validation studies of the Daily Activity Behaviours Questionnaire (DABQ). A total of 268 participants (143 females; age range between 15 and 81 years) provided self-reported (DABQ) and device-measured (accelerometer activPAL) estimates of 24-hour movement behaviours, and their socio-demographic information. We used a set of multiple linear regression models to develop calibration equations based on 70% of the sample (e.g. dependent variable: device-measured SB; independent variables: self-reported SB, MVPA, and sleep, age, sex, body mass index) and conducted cross-validation on the remaining 30% of the sample.

**Results:**

Self-reported MVPA, LPA, SB, and sleep explained 22%, 18%, 24%, and 41% of the variance of device-measured MVPA, LPA, SB, and sleep, respectively. When adding age, sex, body mass index, and other two self-reported movement behaviours to the model, explained variance improved to 31%, 36%, 37%, and 42%, respectively. Non-calibrated self-reported estimates on SB (-110 min/day, 95% CI: -147, -74) and MVPA (-31 min/day, 95% CI: -39, -23) showed underestimation, LPA (136 min/day, 95% CI: 100, 172) overestimation, and sleep time no significant difference (5 min/day, 95% CI: -6, 16). However, we observed no significant difference (± 2 min/day) between calibrated self-reported and device-measured movement behaviours, with substantially narrower limits of agreement.

**Conclusion:**

Our results showed that calibrated DABQ provide comparably accurate group-level estimates of 24-hour movement behaviours than accelerometer activPAL. Questionnaire calibration is a relatively simple method to improve the validity of self-reported estimates that have been underutilized in physical activity research to date. Improved validity of calibrated self-reported estimates on 24-hour movement behaviours may benefit epidemiological research, population surveillance systems, and individual-level assessments in practice.

